# Efficacy of Dupilumab in Treating Atopic Dermatitis With Recurrent Eczema Herpeticum in a Patient With DOCK8-Deficiency Hyper-IgE Syndrome: A Case Report

**DOI:** 10.7759/cureus.43360

**Published:** 2023-08-12

**Authors:** Reshale A Johar, Afnan Hasanain, Yousef Khouqeer

**Affiliations:** 1 Medicine, King Saud Bin Abdulaziz University for Health Sciences, Jeddah, SAU; 2 Dermatology, King Faisal Specialist Hospital and Research Centre, Jeddah, SAU

**Keywords:** dock8-deficiency, dupilumab, herpes, eczema, hyper-ige syndrome

## Abstract

Dupilumab, a monoclonal antibody targeting interleukin 4 and interleukin 13, was used to successfully induce remission of chronic, disseminated eczema herpeticum in a six-year-old girl who has DOCK8-deficiency hyper-IgE syndrome. The patient was started on 200 mg of dupilumab administered once every four weeks. The patient had achieved complete resolution of all active herpetic lesions by the time her third dose was due. During the course of three months, she had not developed any new lesions, and significant improvement of the patient’s skin, scalp, hair restoration, and nails was appreciated.

## Introduction

Hyper-immunoglobulin E syndrome (HIES) is a rare immunodeficiency disorder with two classifications: autosomal-dominant hyper-IgE syndrome (AD-HIES) caused by a signal transducer and activator of transcription 3 (STAT3) gene mutation, and autosomal-recessive hyper-IgE syndrome (AR-HIES) caused by mutations in either dedicator of cytokinesis 8 (DOCK8), PGM3, TKY2, and SPINK5 genes [1,2]. Although the incidence of HIES is less than 1/100,000, it is more prevalent in regions with consanguineous populations [3,4].

The two classifications of HIES share common clinical presentations of dermatitis, recurrent infections (i.e., staphylococcal, candidiasis, respiratory tract infections), autoimmunity, viral-based malignancies, eosinophilia, and elevated serum-IgE levels. However, patients with AR-HIES present with more severe atopic complications, including food allergies, asthma, eczema, and mucocutaneous bacterial and viral infections (i.e., eczema herpeticum), which are considered less common in AD-HIES patients [4,5]. Distinguishably, the viral infections AR-HIES patients persistently suffer from include Varicella zoster, Herpes simplex, and Human Papilloma viruses [6,7]. These infections are usually found in conjunction with recurring cutaneous bacterial infections, particularly Staphylococcus aureus [8].

Besides the important role of the DOCK8 protein in dendritic cell migration and cytoskeletal organization, its deficiency leads to detrimental reduction of natural killer cell (NK cell) toxicity, continuous germinal center B-cells, and the early apoptosis of T-lymphocytes [9]. These implications on cellular and humoral immunity significantly decrease the mature T-cells and memory B-cell reservoirs, respectively. Subsequently, contributing to the immunodeficiency-associated manifestations of AR-HIES; particularly, recurring infections [10]. 

## Case presentation

**Figure 1:**
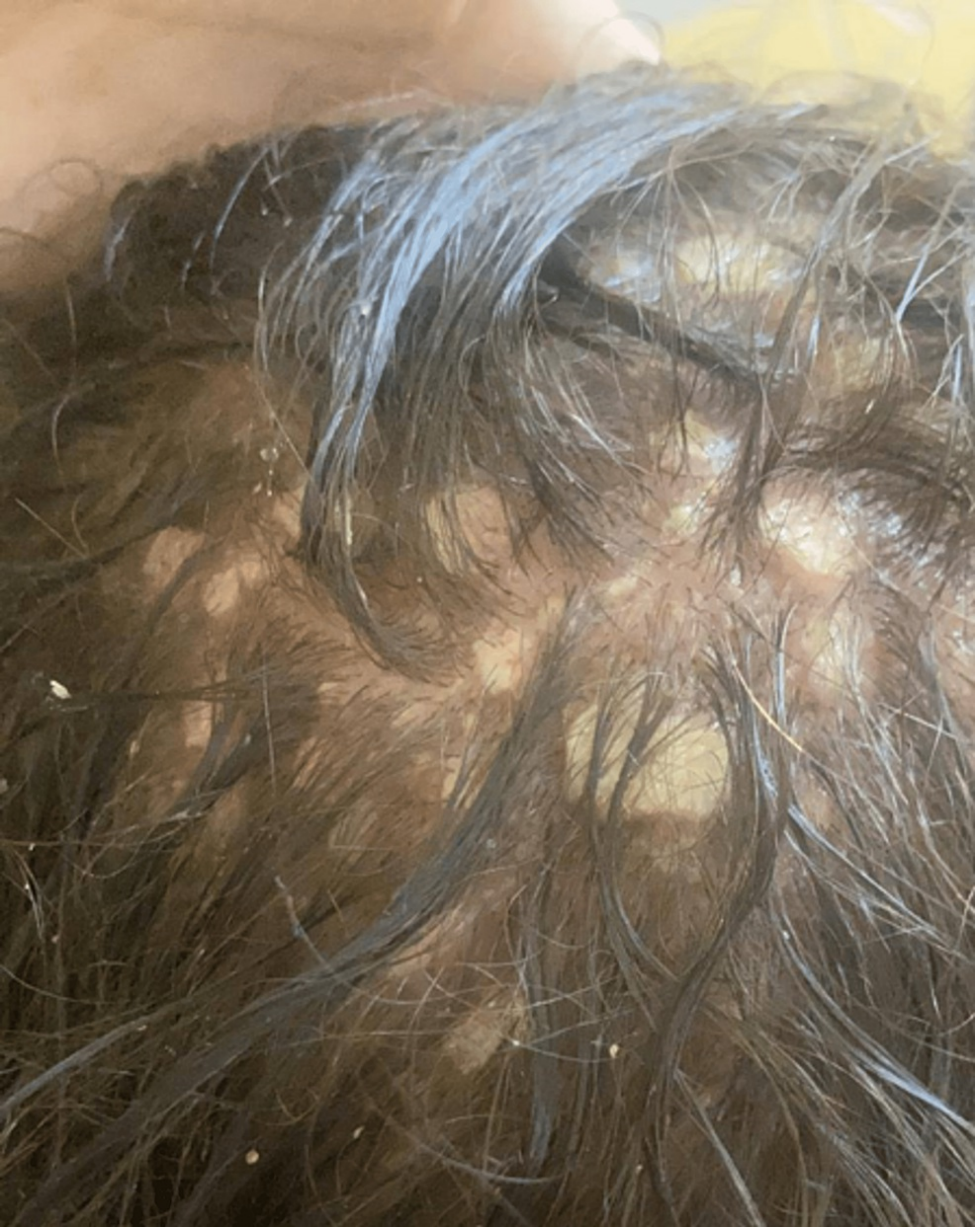
patient’s scalp before receiving dupilumab

**Figure 2:**
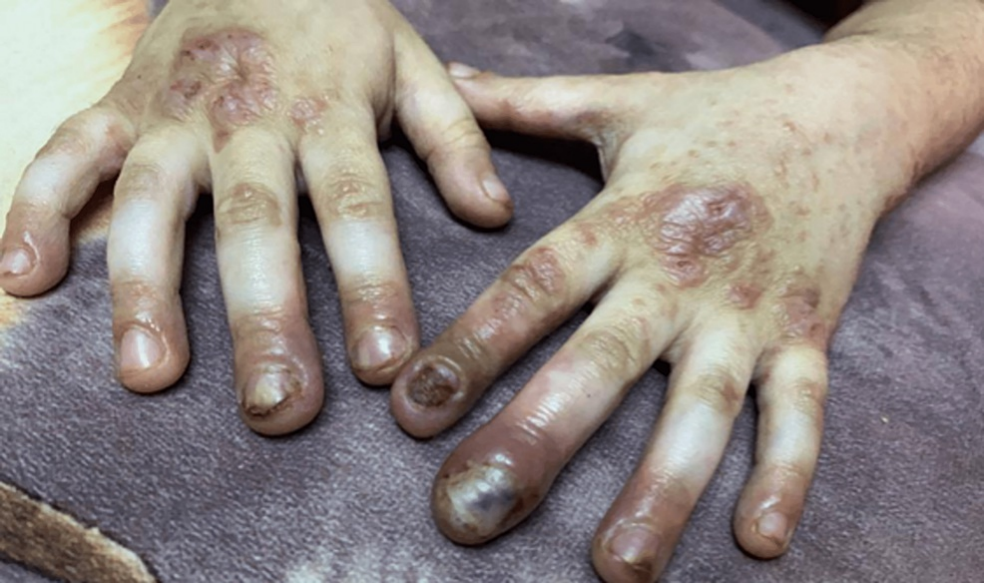
patient’s nails before receiving dupilumab

**Figure 3:**
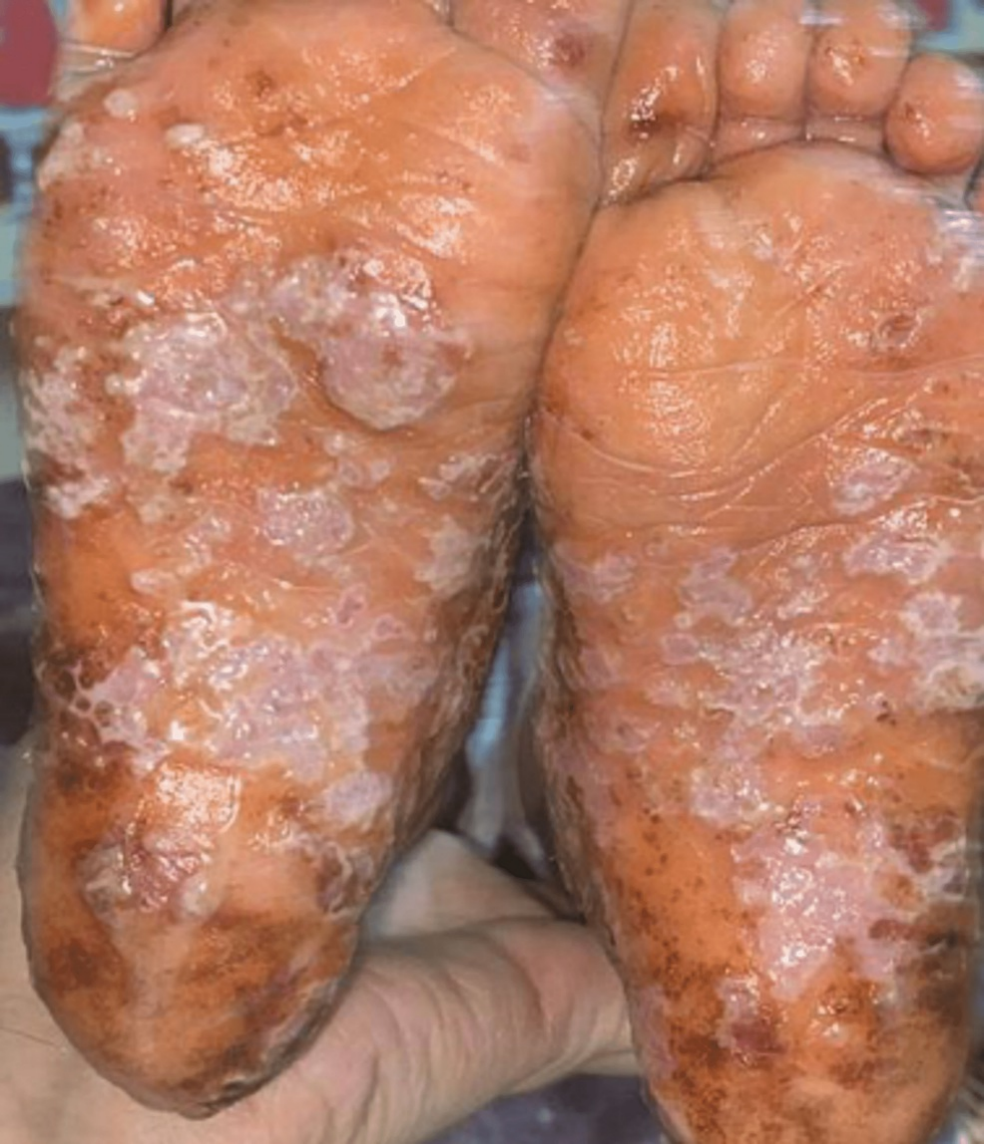
patient’s soles before receiving dupilumab

A six-year-old girl who is the offspring of a consanguineous marriage had gone genetic testing as she was constantly suffering from severe disseminated and recurrent infections during her first years of life without any obvious explanation. She was then referred to our facility to seek treatment. Genetic testing then revealed she had hyper-IgE syndrome with DOCK 8 mutation. Upon presentation, she was complaining of atopic dermatitis with widely disseminated herpetic skin eruption with superimposed bacterial infection involving the face, trunk, back, and the distal ends of all four extremities. Pustules of varying sizes covered the scalp causing secondary hair loss (Figure 1). She also had bilateral well-defined hyperpigmented plaques laced with mildly excoriated vesicles and pustules on the distal ends of both arms and legs from the knee below. Darkening and thickening of the fingertips were noted along with subungual keratosis, onychodystophy, and bluish discoloration of the nail plate. There was also lichenification and excoriation of the skin overlying her metacarpophalangeal joints (Figure 2). The plantar surface of both feet had various patches of skin sloughing and ulceration (Figure 3). Since infancy, she has been admitted numerous times for sinopulmonary infections, multiple episodes of gastroenteritis, and chronic lymphadenopathy. She has also had recurrent herpes stomatitis and eczema herpeticum with a superimposed bacterial infection that would only transiently respond to monthly intravenous immunoglobulin (IVIG) transfusions, intravenous (IV) acyclovir, antibiotics, and topical steroids. There is a positive family history of hyper-IgE syndrome affecting her sister and distant female cousin.

**Figure 4:**
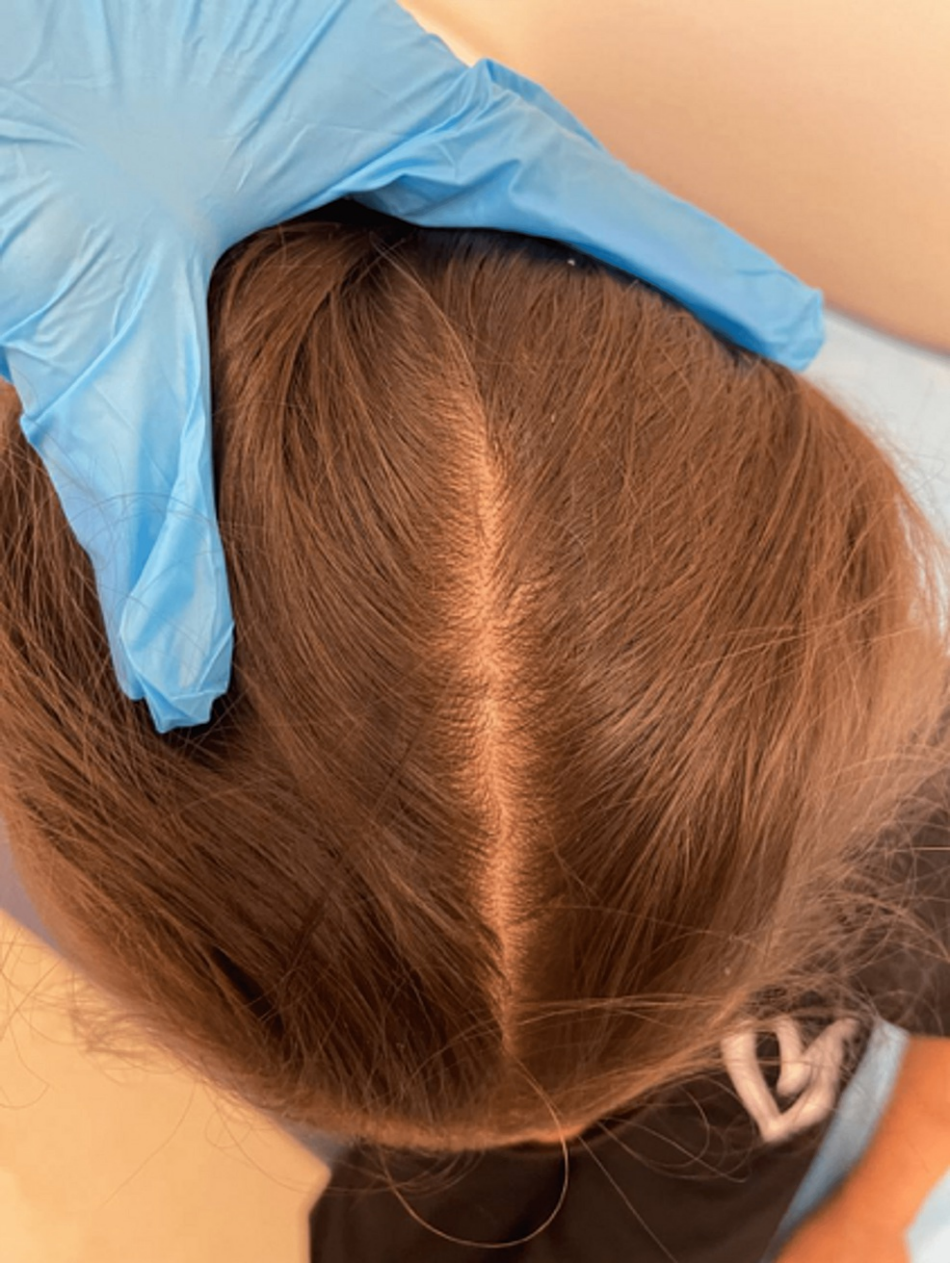
scalp showing lesion resolution and hair regrowth after dupilumab

**Figure 5:**
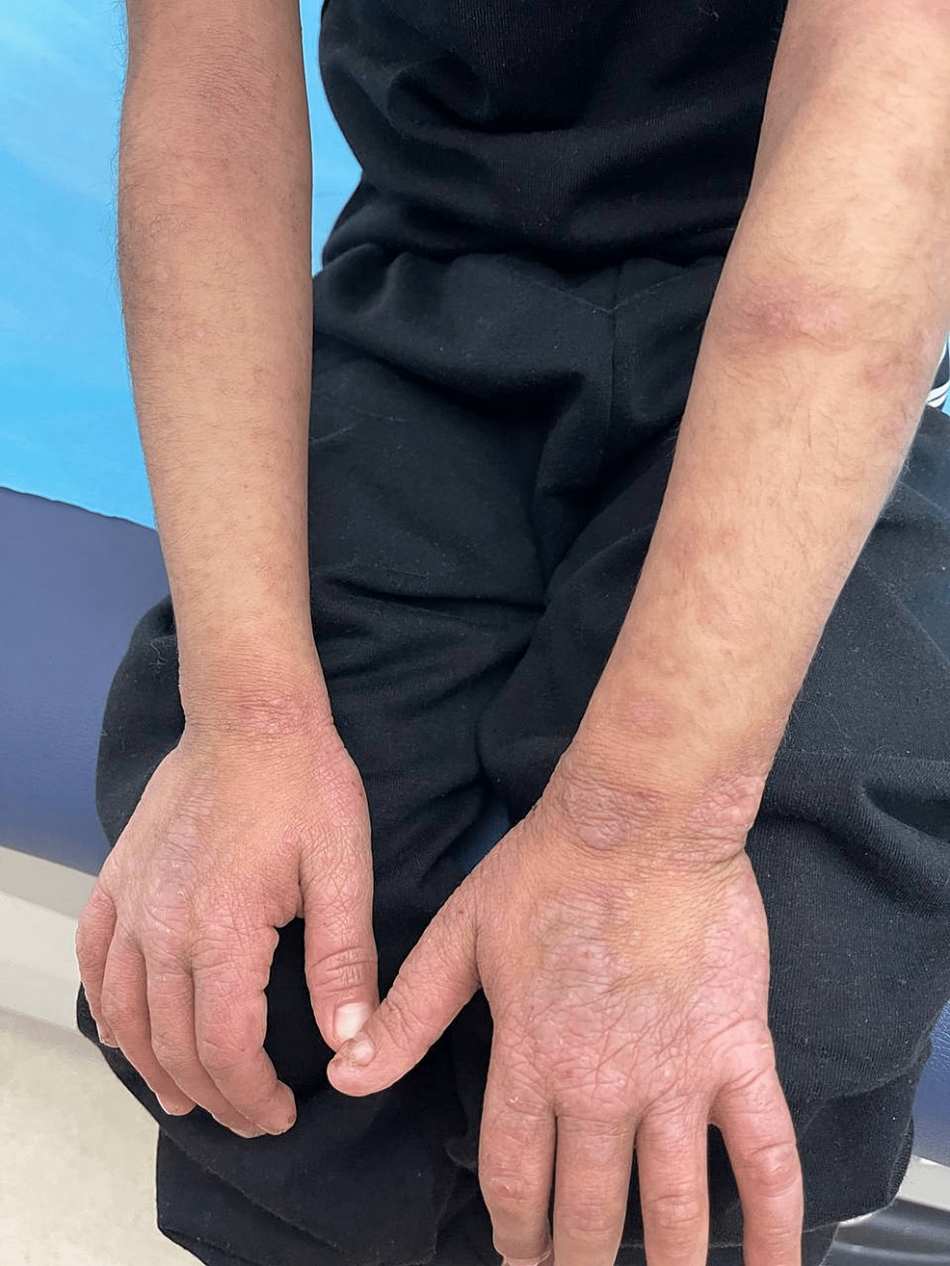
nail regrowth and absence of active lesions after initiating dupilumab

**Figure 6:**
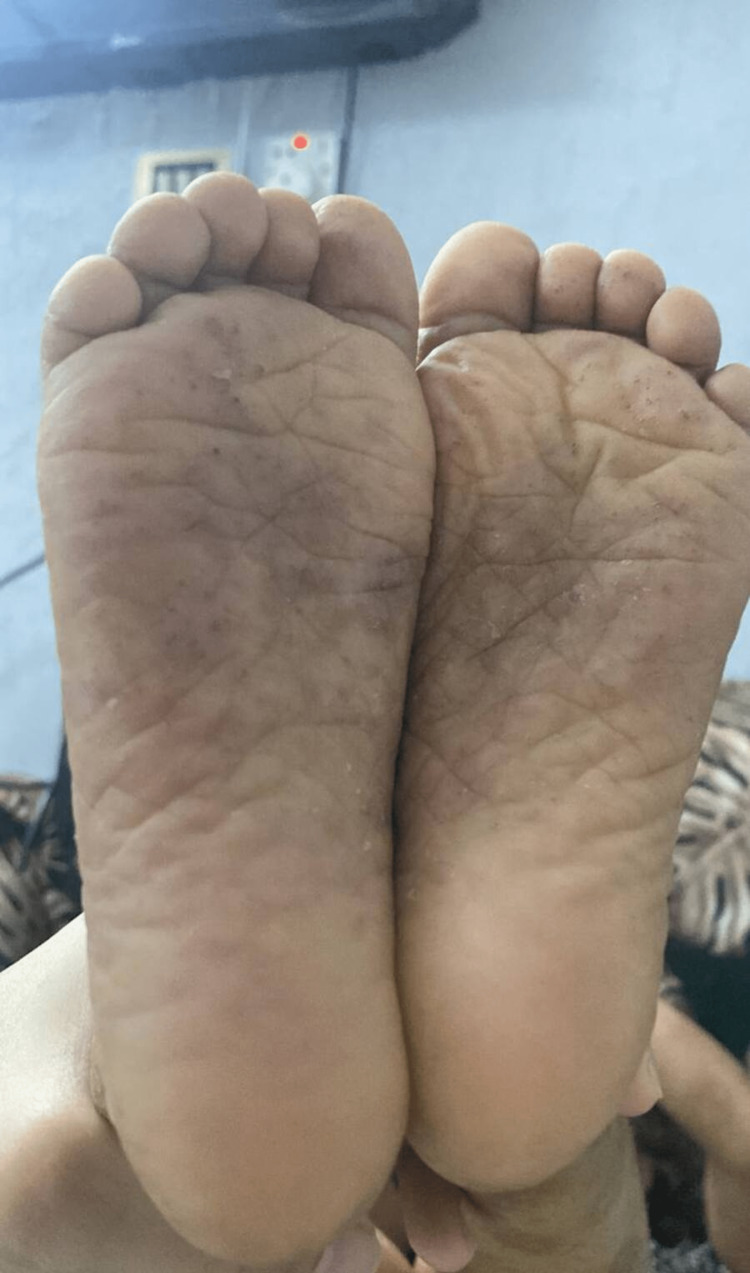
skin regeneration and absence of ulcers after commencing dupilumab

The patient was started on subcutaneous dupilumab 200 mg every four weeks. After the administration of three doses, marked improvement was seen. There was complete resolution of the pustular scalp lesions and new healthy hair growth with no residual alopecic patches or fibrosis (Figure 4). Residual skin thickening, mild xerosis, and hyperlinearity were noted on all the extremities along with healthy nail regrowth (Figure 5). All former abrasions and open wounds had healed including the ulceration on both soles of the feet (Figure 6). Asymptomatic hypopigmented patches with hyperpigmented borders were seen on the trunk, back, and buttocks, and no active lesions were found. No new lesions were reported nor were identified on examination. She is scheduled to receive her fourth dose later this month and continue with this regimen alongside regular until complete remission is achieved and maintained. The mother reports that her daughter stopped developing new lesions after the first dose of dupilumab. 

## Discussion

The lack of randomized-treatment trials available to help structure the proper treatment protocol in such cases is mainly attributed to the low prevalence of HIES. Its management mainly hinges on available clinical experience and observational data [11]. Thus the main goals when treating AR-HIES patients focus on treating dermatological conditions, such as eczema and pruritus, and the management/ prevention of severe systematic infections. Atopic conditions are considered the most prevalent clinical manifestation of AR-HIES DOCK8 deficiency [6]. The elevation of serum-IgE levels correlates with the heightened hypersensitivity immune responses seen in AR-HIES patients, particularly eczematoid dermatitis [4]. Hence, identifying any underlying dermatological conditions that may exacerbate inflammation and cutaneous infections, respectively, becomes imperative in the proper management and treatment of patients with this condition.

Over the years, increasing phenotypic varieties have been identified as the underlying cause of AR-HIES; however, the majority of cases are due to a loss-of-function mutation in DOCK8 [12-14]. This deficiency elicits a cascade of cellular and humoral changes which impact the immune system, namely: the production of defective NK T-cells, CD8 T-cells, and the impaired function and generation of Th17-cells, NK cells, and antiviral cytokines [13,14]. These consequences in conjunction with the cytokine secretion abnormalities and severe decrease in the production of IL-2, TNF-α, and IFN-γ commonly seen in AR-HIES patients [15,16], result in a strong skewing towards the production of Th2 CD4+ T-cell [17]. This Th2 subset is responsible for the secretion of interleukin-4 (IL-4) and interleukin-5 (IL-5) cytokines, which play major roles in the regulation of lymphocyte proliferation, and atopic and allergic disease. Subsequently, framing the overproduction IL-4 and IL-5 as the underlying factors behind the dermatological manifestations of AR-HIES [18].

Dupilumab is a human monoclonal antibody that acts as an interleukin-4 (IL-4) receptor alpha antagonist [19]. The IL-4 receptor alpha subunit is shared by the receptor complexes of IL-4 and interleukin-13 (IL-13). Subsequently, blocking the action of the IL-4 receptor alpha subunit allows duplimab to repress IL-4 and IL-13 mediated responses, including the release of proinflammatory chemokines, cytokines, and most importantly immunoglobulin E (IgE). Inhibiting this proinflammatory cascade has been observed to diminish eczematous dermatitis and associated dermatological manifestations in patients with AR-HIES. Similarly, omalizumab is a human recombinant monoclonal antibody against IgE that ultimately mitigates the inflammatory cascade response by reducing IgE serum levels [11]. It has been previously used to successfully treat HIES patients’ dermatological symptoms by inhibiting the IgE-mediated inflammatory cascade [11,20]. Comparable results are seen in this case with the administration of dupilumab to a HIES patient. While it has demonstrated a range of therapeutic results in this case, prospective studies alongside long-term follow-ups are essential to fully understand the extent and efficacy of dupilumab as a treatment for HIES.

## Conclusions

The clinical treatment of AR-HIES patients must include supportive care such as emollient creams, antihistamines, and antibiotics in the presence of infection as they navigate living with its manifestations of recurrent cutaneous infections, allergies, eosinophilia, elevated IgE levels, and the dermatological manifestations of eczema, dermatitis, and pruritus. Although there is yet to be a cure-all for HIES, anti-IgE therapy with dupilumab should be considered as a viable adjunctive treatment in similar cases.
